# Induction of apoptosis by sarijang, a bamboo salt sauce, in U937 human leukemia cells through the activation of caspases

**DOI:** 10.3892/etm.2013.1151

**Published:** 2013-06-10

**Authors:** EUN-A CHOI, CHEOL PARK, MIN-HO HAN, JUN HYUK LEE, GI-YOUNG KIM, BYUNG TAE CHOI, YUNG HYUN CHOI

**Affiliations:** 1 Insan Bamboo Salt Inc. and Insan Oriental Medical Clinic, Hamyang, Gyeongsangnam-do 676-805;; 2 Department of Molecular Biology, College of Natural Sciences, Dongeui University, Busan 614-714;; 3 Department of Biochemistry, Dongeui University College of Oriental Medicine, Busan 614-052;; 4 Anti-Aging Research Center and Blue-Bio Industry RIC, Dongeui University, Busan 614-714;; 5 Biotechnology Examination Division, Chemistry and Biotechnology Examination Bureau, Korean Intellectual Property Office, Daejeon 302-701;; 6 Department of Marine Life Sciences, Jeju National University, Jeju 690-756;; 7 Division of Meridian and Structural Medicine, School of Korean Medicine, Pusan National University, Yangsan, Gyeongsangnam-do 626-870, Republic of Korea

**Keywords:** sarijang, U937, apoptosis, caspases

## Abstract

Sarijang is a bamboo salt soy sauce, containing extracts of *Rhynchosia nulubilis*, sulfur-fed duck, dried bark of *Ulmus davidiana* and *Allium sativum*, which has been demonstrated to exert anti-inflammatory and antitumor activity. However, the cellular and molecular mechanisms of action of sarijang have not yet been elucidated. In the present study, we investigated the pro-apoptotic effects of sarijang in an *in vitro* U937 human leukemia cell model. Treatment with sarijang resulted in a concentration-dependent growth inhibition of the cells, coupled with the characteristic morphological features of apoptosis. The induction of the apoptotic cell death of the U937 cells by sarijang exhibited a correlation with the upregulation of death receptor 4 (DR4), the downregulation of members of the inhibitor of apoptosis protein (IAP) family, including survivin and cellular IAP (cIAP)-1, and the cleavage of Bid. Apoptosis-inducing concentrations of sarijang also induced the activation of caspases (caspase-3, -8 and -9), accompanied by proteolytic degradation of poly(ADP-ribose)-polymerase, β-catenin and phospholipase C-γ1. However, the apoptosis induced by sarijang was significantly inhibited by z-VED-fmk, a pan-caspase inhibitor, which demonstrated the importance of caspases in the process. These results suggested that sarijang may be a potential chemotherapeutic agent for use in the control of U937 human leukemia cells. Further studies are required to identify the active compounds in sarijang.

## Introduction

Leukemia, a malignant hematopoietic tumor, is a cancer of the blood or bone marrow that is characterized by the abnormal proliferation of white blood cells. It is the sixth highest ranking variety of human tumor worldwide (1). Leukemias are classified into acute lymphocytic, originating in the lymphocytes in bone marrow, and myelogenous leukemia, originating from granulocytes or monocytes (2,3). However, leukemia is highly resistant to chemotherapy, and there is no effective cure for patients in the advanced stages of the disease. To overcome these challenges, novel therapeutic strategies are required for the more efficacious treatment of this disease. Moreover, effective chemopreventive treatments for leukemia are likely to have a significant impact on leukemia morbidity and mortality.

Apoptosis is an active process of programmed cell death that has been characterized as a fundamental cellular activity to maintain the physiological balance of the organism. In general, apoptosis is mediated through two major pathways, the extrinsic [death receptor (DR)-mediated] and intrinsic (mitochondrial-mediated) pathways (4,5). The products of several genes have been demonstrated to be critical in the regulation of apoptosis, including the Bcl-2 and inhibitor of apoptosis protein (IAP) family members and the caspase cascades. In addition, apoptosis is involved in the immune defense machinery, and functions as a protective mechanism against carcinogenesis by eliminating damaged cells or abnormal excess cells that proliferate due to the induction of various chemical agents (6–8). A number of investigations have indicated that the induction of apoptosis in tumor cells is the most common anti-cancer mechanism, utilized by numerous cancer therapies. Therefore, the induction of apoptotic cell death by certain chemotherapeutic agents is an important mechanism in the anti-cancer properties of many drugs.

Sarijang is a bamboo salt soy sauce that is made by fermenting *Rhynchosia nulubilis*, a plant that exhibits potent detoxifying properties, boiling it with sulfur-fed duck, *Ulmus davidiana* var. *japonica* Nak., *Allium sativum* (garlic) and sap of the lacquer tree, mixing this combination with bamboo salt and then aging the mixture, as previously described (Choi E-A: A method for producing healthful soy sauce. Korean Patent. Filed: July 30, 2004; issued: June 13, 2005). It has been suggested that sarijang may exert medicinal effects due to the fact that bamboo salt, which is a major raw material of sarijang, is known to exhibit anti-inflammatory and anti-cancer effects (9–13). *R. nulubilis* contains high levels of genistin, daidzin, genistein and daidzein, which are isoflavones present in general beans, in addition to high levels of aglycone, existing in a state that is not bound to glycoside. Therefore, *R. nulubilis* is expected to possess anti-cancer, anti-inflammatory and immunity-enhancing effects, as well as being effective in the prevention of menopausal osteoporosis (14–16). Furthermore, the dried bark of *U. davidiana* has been demonstrated to be highly effective at protecting against cytotoxicity and preventing osteoporosis and asthma, in addition to exerting anti-inflammatory and immunity-boosting effects (17–19). Garlic, which is a source of sulfur-containing compounds (20–22), and sulfur-fed duck extract have been revealed to possess excellent anti-inflammatory and immunity-boosting properties, as well as anti-cancer or cancer-preventing effects. Although the components of sarijang have yet to be analyzed, the results of previous analyses of the major raw materials of sarijang suggest that sarijang may exhibit the effects demonstrated by each of the individual diverse components. However, there is insufficient scientific evidence to support this. Therefore, the aim of the present study was to examine the anti-cancer effects of sarijang, as part of an investigation into its medicinal efficacy. As such, we evaluated whether sarijang was able to inhibit cell growth and induce apoptosis in an *in vivo* U937 human leukemia cell model.

## Materials and methods

### Reagents and antibodies

Fetal bovine serum (FBS), RPMI-1640, penicillin, streptomycin and trypsin-EDTA were purchased from Gibco-BRL (Gaithersburg, MD, USA). 4′,6-Diamidino-2-phenylindole (DAPI), propidium iodide (PI), paraformaldehyde, 3-(4,5-dimethyl-2-thiazolyl)-2,5-diphenyl-2H-tetrazolium bromide (MTT), RNase A and proteinase K were obtained from Sigma-Aldrich (St. Louis, MO, USA), and an enhanced chemiluminescence (ECL) kit was purchased from Amersham Corp. (Arlington Heights, IL, USA). Caspase activity assay kits were obtained from R&D Systems, Inc. (Minneapolis, MN, USA) and the pan-caspase inhibitor, z-VED-fmk, was obtained from Calbiochem-Novabiochem Corp. (San Diego, CA, USA). DNA ladder size markers were purchased from Invitrogen Life Technologies (Carlsbad, CA, USA), while the antibodies of tumor necrosis factor-related apoptosis-inducing ligand (TRAIL), DR4, DR5, Fas, Fas ligand (FasL), X-linked IAP (XIAP), cellular IAP (cIAP)-1, cIAP-2, survivin, Bcl-2, Bcl-xL, Bax, Bad, Bid, caspases-3, -8 and -9, poly(ADP-ribose)-polymerase (PARP), β-catenin, phospholipase C-γ1 (PLC-γ1) and actin were purchased from Santa Cruz Biotechnology, Inc., (Santa Cruz, CA, USA). Horseradish peroxidase (HRP)-conjugated anti-mouse and anti-rabbit secondary antibodies were obtained from Amersham Corp, while any additional chemicals not specifically cited here were purchased from Sigma-Aldrich.

### Cell culture and treatment of sarijang

The U937 human leukemia, Chang liver and WI-38 (an immortalized non-tumor cell line derived from normal human liver tissue and an embryonic lung fibroblast, respectively) cells were purchased from the American Type Culture Collection (Rockville, MD, USA) and maintained at 37°C in 95% humidified air and 5% CO_2_ in RPMI-1640 supplemented with 10% heat-inactivated FBS, 2 mM glutamine, 100 U/ml penicillin, and 100 *μ*g/ml streptomycin. Sarijang was provided by Insan Bamboo Salt Inc. (Hamyang, Republic of Korea) and was filtration sterilized using 0.4-*μ*l single filters, and diluted with medium to the desired concentration prior to use.

### Cell proliferation assay

Cells were seeded into 6-well plates at a density of 1×10^5^ cells/ml and incubated for 24 h at 37°C, with the absence and presence of variable concentrations of sarijang. Following incubation, cells were washed with phosphate-buffered saline (PBS), trypsinized and manually counted with a hemocytometer through the exclusion of trypan blue. For the morphological study, the cells were treated with sarijang for 24 h and then photographed directly using an inverted microscope (Carl Zeiss, Oberkochen, Germany).

### Cell viability assay

The cell viability assay was performed using an MTT assay. For the MTT assay, cells were treated with sarijang for 24 h. Following the treatments, 0.5 mg/ml MTT solution was added, prior to incubation for 2 h at 37°C in the dark. The absorbance of each well was measured at 540 nm with an enzyme-linked immunosorbent assay (ELISA) reader (Molecular Devices, LLC, Sunnyvale, CA, USA).

### Nuclear staining with DAPI

For DAPI staining, the cells were washed with PBS and fixed with 3.7% paraformaldehyde (Sigma-Aldrich) in PBS for 10 min at room temperature. The fixed cells were then washed with PBS and stained with 2.5 *μ*g/ml DAPI solution for 10 min at room temperature, prior to being washed twice with PBS and analyzed using a fluorescence microscope (Carl Zeiss) (23).

### DNA fragmentation assay

Following sarijang treatment, cells were lysed in a buffer containing 10 mM Tris-HCl (pH 7.4), 150 mM NaCl, 5 mM EDTA, and 0.5% Triton X-100 for 1 h at room temperature. The lysates were vortexed and cleared by centrifugation at 19,000 × g for 30 min at 4°C. A 25:24:1 (v/v/v) equal volume of neutral phenol : chloroform : isoamyl alcohol was used for the extraction of the DNA in the supernatant, followed by electrophoretic analysis on 1.5% agarose gels containing 0.1 *μ*g/ml ethidium bromide (EtBr).

### DNA flow cytometric analysis

Following treatment with sarijang, cells were harvested, washed twice with ice-cold PBS, and fixed with 75% ethanol at 4°C for 30 min. The DNA content of cells was then stained using a CycleTest™ Plus DNA staining kit (Becton Dickinson, San Jose, CA, USA) with PI. The cellular DNA content at the sub-G1 phases was subsequently determined using a FACSCalibur™ flow cytometer (BD Biosciences, Franklin Lakes, NJ, USA), prior to being analyzed with Cell Quest software (Becton Dickinson).

### Protein extraction and western blot analysis

Cells were lysed with lysis buffer [20 mM sucrose, 1 mM EDTA, 20 *μ*M Tris-Cl (pH 7.2), 1 mM dithiothreitol (DTT), 10 mM KCl, 1.5 mM MgCl_2_, 5 *μ*g/ml pepstatin A, 10 *μ*g/ml leupeptin and 2 *μ*g/ml aprotinin] containing protease inhibitors. A Bio-Rad protein assay (Bio-Rad, Hercules, CA, USA) was used in accordance with the manufacturer’s instructions, in order to determine the protein concentrations. Following normalization, an equal quantity of protein was subjected to electrophoresis on sodium dodecyl sulfate (SDS)-polyacrylamide gels, and then transferred to nitrocellulose membranes (Schleicher & Schuell Bioscience, Inc., Keene, NH, USA) by electroblotting. The membranes were blocked with 5% skimmed milk and subsequently incubated with the primary antibodies and the HRP-conjugated anti-mouse or anti-rabbit secondary antibodies. An ECL detection system was used to visualize the target proteins (Amersham Corp.).

### In vitro caspase activity assay

The activity of the caspases was determined using colorimetric assay kits, which utilized the following synthetic tetrapeptides, labeled with *p*-nitroaniline (pNA): Asp-Glu-Val-Asp (DEAD) for caspase-3, Ile-Glu-Thr-Asp (IETD) for caspase-8 and Leu-Glu-His-Asp (LEHD) for caspase-9. In brief, the sarijang-treated and untreated cells were lysed in the supplied lysis buffer. The supernatants were then collected and incubated with the supplied reaction buffer containing DTT and DEAD-, IETD- or LEHD-pNA as substrates at 37°C. The reactions were measured by changes in the absorbance at 405 nm, using the VERSAmax tunable microplate reader (Molecular Devices, PaloAlto, CA, USA) (24).

### Statistical analysis

Unless otherwise indicated, the data are expressed as the mean ± standard deviation of the results obtained from three separate experiments. Statistical analysis was performed using a paired Student’s t-test. P<0.05 was considered to indicate a statistically significant difference.

## Results

### Sarijang inhibits proliferation and cell viability in U937 cells

**Figure 1.**
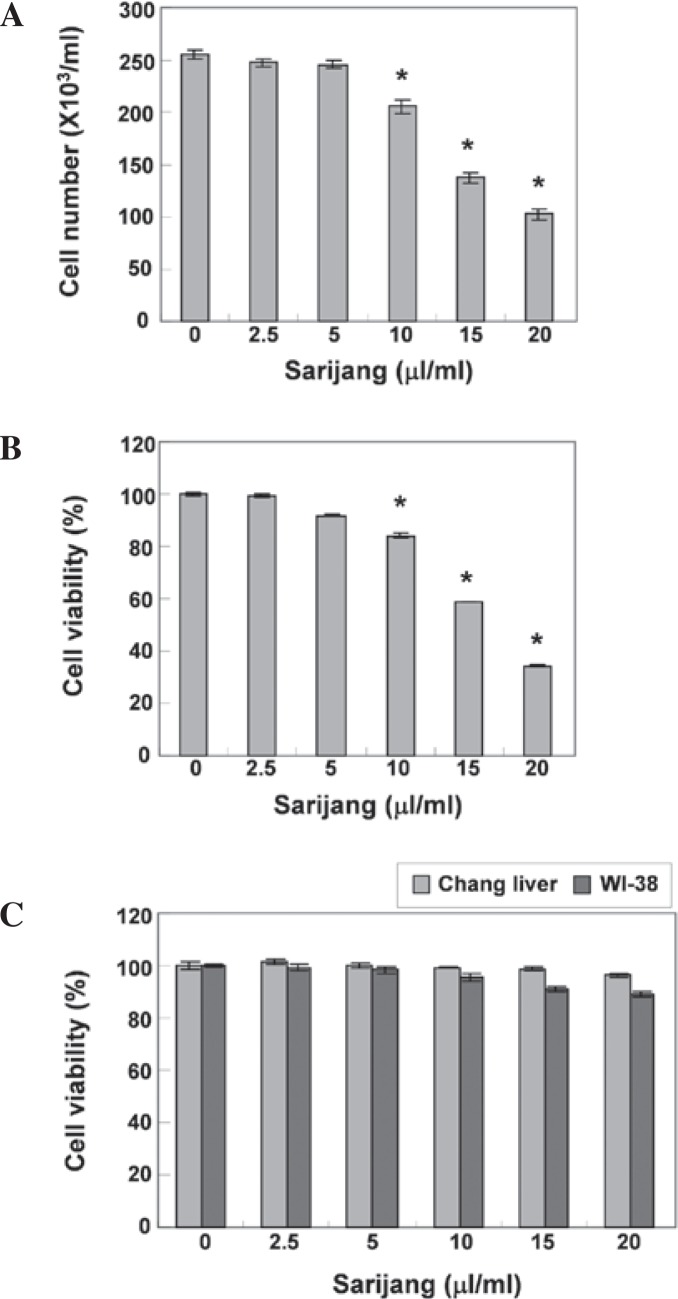
Inhibition of cell proliferation and viability by sarijang in U937 human leukemia cells. Cells were seeded into 6-well plates at a density of 1×10^5^ cells/ml and treated with the indicated concentrations of sarijang for 24 h. The (A) cell number and (B) viability of U937 cells were determined by hemocytometer counts through the exclusion of trypan blue and MTT assay, respectively. (C) The viability of Chang liver and WI-38 lung normal cells was measured using an MTT assay. The results are expressed as percentages of the vehicle-treated control ± standard deviation of three separate experiments. A Student’s t-test was used to determine significance. ^*^P<0.05 vs. untreated control.

**Figure 2.**
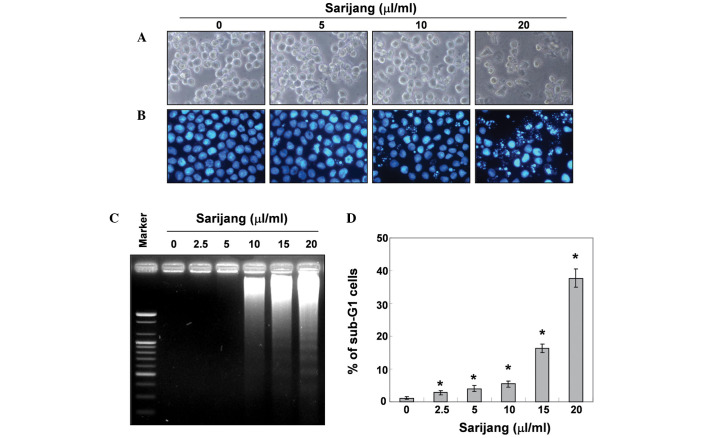
Induction of apoptosis by sarijang treatment in U937 cells. Cells were seeded into 6-well plates at a density of 1×10^5^ cells/ml and treated with the indicated concentrations of sarijang for 24 h. (A) Cell morphology was visualized by light microscopy; magnification, ×200. (B) Nuclei stained with 4’,6-diamidino-2-phenylindole (DAPI) solution were photographed with a fluorescence microscope using a blue filter; magnification, ×400. (C) For analysis of DNA fragmentation, genomic DNA was extracted, electrophoresed in a 1.5% agarose gel and visualized using ethidium bromide (EtBr) staining. (D) To quantify the degree of apoptosis induced by sarijang, cells were evaluated for sub-G1 DNA content, which represented the fractions undergoing apoptotic DNA degradation, using a flow cytometer. The results are expressed as percentages of the vehicle-treated control ± standard deviation of three separate experiments. A Student’s t-test was used to determine significance. ^*^P<0.05 vs. untreated control.

To investigate the effects of sarijang on U937 cell growth, the cells were treated with various concentrations of sarijang for 24 h, and the cell number and viability were then measured by the trypan blue exclusion method and MTT assay, respectively. As demonstrated in Fig. 1A and B, sarijang markedly inhibited the cell proliferation and viability of the U937 cells in a concentration-dependent manner. The cell viability was inhibited by >41 or 63% in cells exposed to 15 or 20 *μ*l/ml sarijang, respectively, as compared with untreated controls. In addition, a visual inspection using inverted microscopy revealed that treatment with sarijang resulted in numerous morphological changes (Fig. 2A). Furthermore, an additional experiment was conducted using Chang liver and WI-38 cells, in order to examine the effect of sarijang on the proliferation of normal cells. The results of this experiment are presented in Fig. 1C. The results demonstrated that the survival rate of Chang liver and WI-38 cells did not significantly change when under the same conditions as those applied to U937 cells.

### Sarijang induces apoptosis in U937 cells

In order to determine whether the growth inhibition by sarijang was associated with the induction of apoptosis, apoptotic features were examined by measuring the chromatin condensation of nuclei, DNA fragmentation and the sub-G1 phase DNA content. As demonstrated in Fig. 2B, treatment with sarijang resulted in the condensation of chromatin in a significant number of cells, and the occurrence of apoptotic body formation in a concentration-dependent manner. These features were not observed in the control cells. In addition, treatment with sarijang induced a progressive accumulation of fragmented DNA, which appeared in a typical ladder pattern of DNA fragmentation. This was due to the internucleosomal cleavage associated with apoptosis, and occurred in a concentration-dependent manner (Fig. 2C). The degree of apoptosis was determined by analyzing the sub-G1 DNA content in the sarijang-treated U937 cells using flow cytometry. As demonstrated in Fig. 2D, treatment with sarijang resulted in an increased accumulation of cells with sub-G1 DNA content. These results revealed a good correlation between the extent of apoptosis and the inhibition of growth in U937 cells.

### Effects of sarijang on the expression of apoptosis-related genes

**Figure 3.**
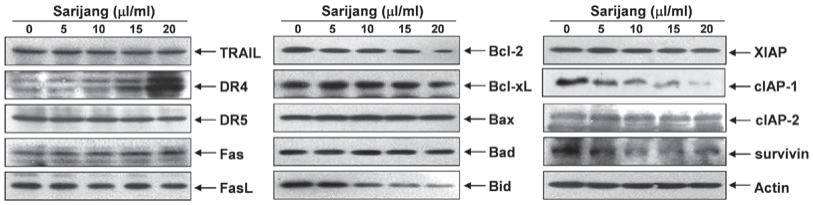
Effects of sarijang on the levels of apoptosis-related genes in U937 cells. Cells were treated with the indicated concentrations of sarijang for 24 h, the cells were lysed and then equal amounts of cell lysates (30–50 *μ*g) were separated on sodium dodecyl sulfate-polyacrylamide gels and transferred to nitrocellulose membranes. Membranes were probed with the indicated antibodies, and the proteins were visualized using an enhanced chemiluminescence detection system. Actin was used as an internal control. TRAIL, tumor necrosis factor-related apoptosis-inducing ligand; DR, death receptor; FasL, Fas ligand; XIAP, X-linked inhibitor of apoptosis protein; cIAP, cellular inhibitor of apoptosis.

In order to determine which apoptosis pathway contributed to sarijang-induced apoptosis, the DR and corresponding pro-apoptotic ligands were examined using western blot analyses. The results revealed that sarijang treatment resulted in a concentration-dependent increase in the levels of DR4, whereas the levels of TRAIL, DR5, Fas and FasL expression remained relatively unchanged in response to sarijang treatment (Fig. 3). The examination of the effects of sarijang on the levels of Bcl-2 family proteins revealed that the levels of anti-apoptotic Bcl-2 proteins were inhibited by sarijang treatment, while the pro-apoptotic protein, Bid, a BH3-only pro-apoptotic member of the Bcl-2 family, was truncated in a concentration-dependent manner (Fig. 3). By contrast, the levels of anti-apoptotic Bcl-xL and pro-apoptotic Bax and Bad remained virtually unchanged in response to sarijang treatment. Under identical conditions, the expression levels of IAP family proteins were also examined. The results of western blotting revealed that sarijang treatment resulted in a concentration-dependent reduction in the expression levels of survivin and cIAP-1, but not XIAP or cIAP-2.

### Sarijang induces the activation of caspases and the cleavage of PARP, β-catenin and PLC-γ1

**Figure 4.**
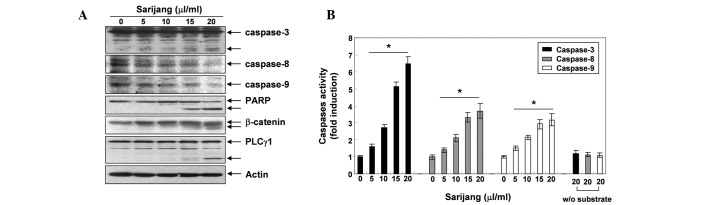
Activation of caspases and the degradation of poly(ADP-ribose)-polymerase (PARP), β-catenin and phospholipase C-γ1 (PLC-γ1) proteins by sarijang treatment in U937 cells. (A) Cells were treated with the indicated concentrations of sarijang for 24 h. The cells were lysed, and then equal amounts of cell lysates (30 *μ*g) were separated on sodium dodecyl sulfate-polyacrylamide gels and transferred to nitrocellulose membranes. Membranes were probed with the indicated antibodies. An enhanced chemiluminescence detection system was used to visualize proteins. Actin was used as an internal control. (B) Cells grown under identical conditions to (A) were collected and lysed. Aliquots were incubated with Asp-Glu-Val-Asp *p*-nitroaniline (DEAD-pNA), Ile-Glu-Thr-Asp (IETD)-pNA, and Leu-Glu-His-Asp (LEHD)-pNA for caspase-3, -8 and -9, individually, at 37°C for 1 h, and the released fluorescence products were measured. The data represent the mean of three independent experiments. A Student’s t-test was used to determine significance. ^*^P<0.05 vs. untreated control.

Following the investigation into the effects of sarijang on the expression of apoptosis-related genes, we examined the expression levels and activities of caspases during sarijang-induced apoptosis. As demonstrated in Fig. 4A, western blotting revealed that the expression levels of the pro-caspases-3, -8 and -9 decreased following sarijang treatment, while levels of the active forms of caspase-3 increased, in a concentration-dependent manner. In addition, the *in vitro* caspase activity in cellular extracts of U937 cells was measured following 24 h exposure to sarijang, using colorimetric substrates specific for each caspase. As illustrated in Fig. 4B, 5–20 *μ*l/ml sarijang significantly stimulated caspase-3, -8 and -9 activities in a concentration-dependent manner. In addition, sarijang treatment led to the progressive proteolytic cleavage of PARP, β-catenin and PLC-γ1 proteins, which are substrates of activated caspase and indicators of apoptosis (Fig. 4A).

### Inhibition of sarijang-induced apoptosis by a pan-caspase inhibitor

**Figure 5.**
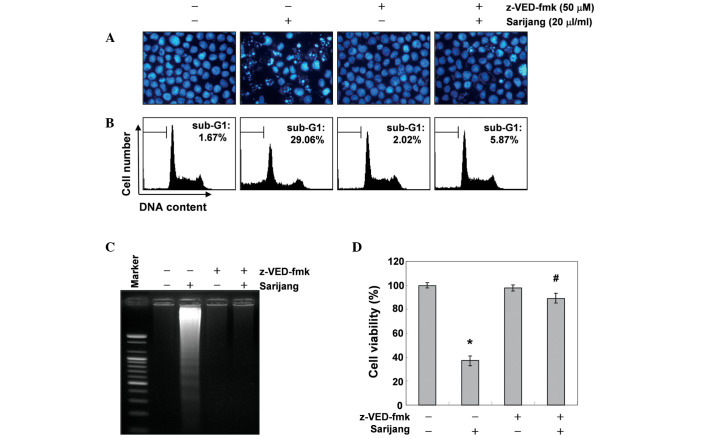
Inhibition of sarijang-induced apoptosis by a pan-caspase inhibitor in U937 cells. U937 cells were pre-treated for 1 h, with or without the pan-caspase inhibitor, z-VED-fmk, and subsequently treated with sarijang for an additional 24 h. (A) Cells were stained with 4′,6-diamidino-2-phenylindole (DAPI) for 10 min and photographed with a fluorescence microscope using a blue filter; magnification, ×400. (B) Cells were evaluated for sub-G1 DNA content using a flow cytometer. The results are expressed as percentages of two separate experiments. (C) Cells grown under identical conditions to (A) were collected, and the genomic DNA was extracted. DNA fragmentation was analyzed by electrophoresis in a 1.5% agarose gel containing ethidium bromide (EtBr). (D) Growth inhibition was measured by the metabolic-dye-based MTT assay. The results are expressed as percentages of the vehicle-treated control ± standard deviation of three separate experiments. A Student’s t-test was used to determine significance. ^*^P<0.05 vs. untreated control; ^#^P<0.05 vs. sarijang-treated cells.

To further confirm the involvement of caspase activation in sarijang-induced apoptotic cell death, cells were pre-treated with or without z-VED-fmk, a pan-caspase inhibitor, for 1 h, followed by treatment with sarijang for 24 h. The results indicated that pre-treatment with z-VED-fmk resulted in a significant prevention of the appearance of cells with apoptotic features, such as chromatin condensation and apoptotic body formation, and an attenuation of the progressive accumulation of fragmented DNA (Fig. 5A and C). Similarly, pre-treatment with z-VED-fmk induced the restoration of decreased cell viability and increased the sub-G1 cell population (Fig. 5B and D). These results indicate that sarijang-induced apoptotic cell death correlated with the activation of caspases in U937 cells.

## Discussion

In the present study, we demonstrated that treatment of a U937 human leukemia cell line with sarijang resulted in the inhibition of cell growth and viability, in addition to changes in cellular morphology, in a concentration-dependent manner (Figs. 1 and 2A). To further confirm that the anti-proliferative effects induced by sarijang were correlated with apoptotic cell death, the levels of chromatin condensation, DNA fragmentation and the induction of the sub-G1 phase were assessed (Fig. 2B–D).

The regulation of apoptosis is critical for the maintenance of development and tissue homeostasis (25). Dysregulated apoptosis is considered to induce a number of pathological conditions, including cancer. Therefore, the induction of apoptosis is an important target for cancer therapy. In general, apoptosis is mediated through two major pathways, the extrinsic and intrinsic pathways. The extrinsic pathway is initiated at the plasma membrane by the binding of DRs to their ligands, such as Fas and FasL, as well as TRAIL and DRs and, subsequently, the activation of caspase-8. Caspase-8, an initiator caspase, is able to directly activate downstream effector caspases, including caspase-3 (4,26,27). The intrinsic pathway is triggered by cell stressors and many chemo-therapeutic agents, resulting in the induction of mitochondrial dysfunction. Mitochondrial dysfunction induces the activation of caspase-9 and subsequently activates effector caspases, such as caspase-3. Following the activation of caspase-3, several specific substrates such as PARP, β-catenin and PLC-γ1 are cleaved, eventually leading to apoptosis (5,6). In certain cells, caspase-8 also mediates the intrinsic pathway via cleavage of the pro-apoptotic Bid protein (6,7). In particular, caspases are known to be regulated by various molecules, including members of the Bcl-2 and IAP families. Bcl-2 family proteins are involved in the control of the apoptotic process by interactions between pro-apoptotic (such as Bax and Bad) and anti-apoptotic (such as Bcl-2 and Bcl-xL) members, particularly those of the intrinsic pathway, leading to mitochondrial dysfunction. Cellular proteins of the IAP family (including XIAP, cIAP-1, cIAP-2 and survivin) specifically inhibit the activity of caspase-3 and -9, while they do not inhibit caspase-8 (4,5). In the intrinsic pathway, members of the IAP family bind directly to the principal caspases, such as pro-caspase-3 and -9, and inhibit the apoptosis induced by Bcl-2 family proteins. Therefore, the downregulation of IAP family proteins relieves the triggering block of proapoptotic signaling and the execution caspases, thus activating cell death (28,29). In the present study, we demonstrated that sarijang markedly upregulated the protein levels of DR4 and induced the cleavage of Bid. In addition, the expression levels of the cIAP-1 and survivin proteins were markedly reduced by sarijang in a concentration-dependent manner (Fig. 3).

Caspases, a family of cysteine-containing aspartate-specific proteases, are known to be important during apoptosis and to lead to the initiation and execution of apoptosis. The activation of initiator caspases, such as caspase-8 and -9, has been demonstrated to result in the downstream activation of effector caspases, such as caspase-3, -6 and -7 (4,5). In particular, the activation of capase-3 is responsible for the proteolytic degradation of numerous key proteins, including PARP and β-catenin, leading to apoptosis (30,31). In the present study, the data indicated that treatment with sarijang induced the activation of capase-3, -8 and -9 and the concomitant proteolytic degradation of PARP, β-catenin and PLC-γ1 proteins (Fig. 4). However, pre-treatment with a pan-caspase inhibitor, z-VED-fmk, prevented the chromatin condensation and DNA fragmentation induced by sarijang and restored cell viability (Fig. 5). The present data demonstrated that sarijang induced an increase in the levels of DR4 and the enzymatic activity of extrinsic and intrinsic caspase cascades, such as caspase-8 and -9, which was correlated with increased levels of truncated Bid expression. In addition, caspase-3 was then activated, and PARP, β-catenin and PLC-γ1 proteins were progressively cleaved in sarijang-treated U937 cells.

In conclusion, the results of this study demonstrated that sarijang triggers the apoptosis of U937 human leukemia cells through the activation of the intrinsic caspase pathway, along with the DR-mediated extrinsic pathway, and that the activation of caspases is responsible for the mediation of sarijang-induced apoptosis. Although the results of this study provide novel information on the possible mechanisms for the anti-cancer activity of sarijang, further studies are required to identify the active compounds.
